# Kinematic Components of the Reach-to-Target Movement After Stroke for Focused Rehabilitation Interventions: Systematic Review and Meta-Analysis

**DOI:** 10.3389/fneur.2018.00472

**Published:** 2018-06-25

**Authors:** Kathryn C. Collins, Niamh C. Kennedy, Allan Clark, Valerie M. Pomeroy

**Affiliations:** ^1^Faculty of Human Science and Public Health, School of Health and Social Sciences, Bournemouth University, Bournemouth, United Kingdom; ^2^School of Psychology, Ulster University, Coleraine, United Kingdom; ^3^Norwich Medical School, University of East Anglia, Norwich, United Kingdom; ^4^Acquired Brain Injury Rehabilitation Alliance, School of Health Sciences, University of East Anglia, Norwich, United Kingdom

**Keywords:** stroke rehabilitation, reaching, upper limb, kinematics, movement performance

## Abstract

**Background:** Better upper limb recovery after stroke could be achieved through tailoring rehabilitation interventions directly at movement deficits.

**Aim:** To *identify potential; targets for therapy by* synthesiz*ing* findings of differences in kinematics and muscle activity between stroke survivors and healthy adults performing reach-to-target tasks.

**Methods:** A systematic review with identification of studies, data extraction, and potential risk of bias was completed independently by two reviewers. Online databases were searched from their inception to November 2017 to find studies of reach-to-target in *people-with-stroke* and healthy adults. *Potential* risk-of-bias was assessed using the Down's and Black Tool. Synthesis *was undertaken via*: (a) meta-analysis of kinematic characteristics utilizing the standardized mean difference (SMD) [95% confidence intervals]; and (b), narrative synthesis of muscle activation.

**Results:** Forty-six studies met the review criteria but 14 had insufficient data for extraction. Consequently, 32 studies were included *in the meta-analysis*. Potential risk-of-bias was low for one study, unclear for 30, and high for one. Reach-to-target was investigated with 618 *people-with-stroke* and 429 healthy adults. *The meta-analysis* found, in all areas of workspace, that *people-with-stroke had*: greater movement times (seconds) e.g., SMD 2.57 [0.89, 4.25]; lower peak velocity (millimeters/second) e.g., SMD −1.76 [−2.29, −1.24]; greater trunk displacement (millimeters) e.g. SMD 1.42 [0.90, 1.93]; *a more curved reach-path-ratio* e.g., SMD 0.77 [0.32, 1.22] and reduced movement smoothness e.g., SMD 0.92 [0.32, 1.52]. In *the* ipsilateral and contralateral workspace, *people-with-stroke exhibited*: larger errors in *target* accuracy e.g., SMD 0.70 [0.39, 1.01]. In contralateral workspace, stroke survivors had: reduced elbow extension and shoulder flexion (degrees) e.g., elbow extension SMD −1.10 [−1.62, −0.58] and reduced shoulder flexion SMD −1.91 [−1.96, −0.42]. Narrative synthesis of muscle activation found that *people-with-stroke, compared with healthy adults*, exhibited: delayed muscle activation; reduced coherence between muscle pairs; and use of a greater percentage of muscle power.

**Conclusions:** This first-ever meta-analysis of the kinematic differences between people with stroke and healthy adults performing reach-to-target found statistically significant differences for 21 of the 26 comparisons. *The differences identified and values provided are potential foci for tailored rehabilitation interventions to improve upper limb recovery after stroke*.

## Introduction

Reaching is essential for everyday activities such as drinking, *using a touch screen or operating buttons on an elevator*. Rehabilitation therefore gives emphasis to regaining reaching ability through evidenced-based task-specific training. *Many people after stroke have upper limb disability, for example: approximately 48% of a consecutive admissions sample at three days after stroke(1); and 65% of individuals with severe stroke not regaining the ability to reach and grasp everyday objects despite participation in rehabilitation (2)*.

There are many different therapy approaches available to clinicians to progress upper limb motor function. An alternative to best conventional therapy is offered by impairment-orientated therapy (3). This impairment-orientated training involves targeting interventions at the movement control deficits underlying difficulty and inability to perform everyday functional tasks. Therefore, a precursor to continuing investigation of impairment-orientated training is to identify the exact movement control deficits experienced by stroke survivors.

Movement control deficits can be identified by kinematic assessment providing sensitive, objective and reliable measurement (4–9). Therefore, kinematic assessment can be used to identify movement control deficits as targets for impairment-orientated training after stroke. Indeed, reaching kinematics has been studied widely in both healthy populations (10–12) and in people after stroke (13–16). Even more information can be gained by combining kinematics with measurement of muscle activity (17). For example, electromyography (EMG) provides neurological measures such as spatial-temporal patterns of muscle activity for enhanced understanding of the movement control (*kinematics and muscle activity*) underlying the performance of everyday tasks (18).

Knowledge of the kinematics of all forms of reaching (4, 19) and more specifically, coordination of reach and grasp components (20), has been drawn together in narrative reviews. These reviews are valuable as they provide an expert overview of the kinematics of reaching activity. However, narrative reviews have potential for bias in at least two aspects: identification of the primary studies included (selection bias); and the possibility that synthesis is influenced by author opinion (expert opinion bias). A robust systematic review is required to minimize the risk of potential bias. In addition, review of the neural components of reaching is required alongside the kinematics.

To understand reaching impairment we need to consider the different forms of reaching required for everyday activity e.g., reach-to-target (operate elevator buttons), reach-to-release (put can on shelf); reach-to manipulate (cut paper with scissors); and reach-to-pull (open cupboard). In addition, reaching activity takes place in many workspace areas including: above the head, behind the trunk and to the contralateral side of the reaching upper limb. Diverse forms of reaching for performance of everyday tasks require the ability to utilize different spatial-temporal patterns of muscle activity and limb segment orientations (4, 19). Indeed, kinematic characteristics vary depending on the reaching task and goal (21, 22). A prerequisite for development of impairment-orientated rehabilitation, therefore, requires knowledge of the movement control deficits underlying difficulty performing everyday reaching tasks to enable therapy to be targeted at what needs to change.

The aims of this systematic review were to: (1) systematically synthesise the differences between individuals with stroke and healthy adults for the *kinematics and muscle activations* of reach-to-target; and (2) determine the potential influence of object location on the differences in *kinematics and muscle activity*. Reach-to-target was chosen because it is the precursor component of most everyday upper limb tasks *and is essential for many daily activities such as a using touch screen (tablet, computer), turning on/off light switch, and using a doorbell, or elevator*.

## Methods

The systematic review methodology was based on guidelines by the Cochrane Collaboration (23). Two reviewers worked independently at each stage: title and abstract screening, full text screening, assessment of potential risk of bias, and data extraction. Each reviewer recorded their assessment on a pre-agreed proforma. If there were disagreements the two reviewers referred to the original document in question. If agreement could not be reached then a third researcher was consulted.

### Searching for studies

The search strategy was developed in collaboration with a research librarian. The search was limited to studies published in the English language. The search terms used included: reaching, upper limb, kinematics, biomechanics, movement analysis, electromyography, and stroke. The terms were a combination of MeSH and non-MeSH terms used as text words. Three online databases were searched: MEDLINE, AMED, and EMBASE; the databases were searched from their inception to November 2017. Due to the differences between databases the search strategy was modified for each individual database; an example of the search strategy used for MEDLINE is in Table 1. In addition, the reference lists of relevant papers were hand searched for potential articles that were not retrieved in the electronic search.

**Table 1:** The search strategy used to search the database MEDLINE as example of electronic searches.

Upper extremity OR arm OR hand
(upper limb).tw
Stroke.tw
“range of motion, articular”/ph
Movement/ph
Muscle, skeletal/ph
Motor skills/ph
arm/ph
Exp Muscle contraction (includes isotonic contraction, isometric contraction and excitation contraction coupling)
(muscle activation OR co?contraction OR motor control).tw
(grasp* OR reach* OR grip* OR pinch* OR limb transport).tw
Exp psychomotor performance (includes motor skills and performance analysis)
Electromyograph* OR transcranial magnetic stimulation OR biomechanics
(co?contraction OR EMG OR motor evoked potential OR biomechanic* OR electromyograph* or kinematic* OR object manipulation).tw
(1) OR (2)
(15)AND (3)
(4) OR (5) …OR (11)
(12) OR (13) OR (14)
(16) AND (17) AND (18)
**Limits**: individuals > 18 years of age; human; English Language

*Tw, text word; ph, physiology*.

### Study eligibility criteria

#### Types of studies

All study designs were included except for single case studies, and reviews. Included studies of people after stroke also needed to investigate healthy adults (control) completing identical reach-to-target tasks.

#### Types of participants

The participants in eligible studies had to be at least 18 years of age. For people after stroke there were no limitations placed on lesion location, time since ictus, or number of strokes. Healthy adult participants needed to have no diagnosis of a neurological or musculoskeletal disorder that could potentially influence movement control or reaching.

#### Types of reaching tasks

Studies were eligible if reaching to a target was assessed with the paretic upper limb of the people after stroke and either upper limb of the healthy adult participants. Specific exclusion criteria were: reach-to-grasp of an object, tapping, tracing, drawing tasks, or reaching with the non-paretic limb (stroke survivors).

#### Types of measures

Eligible studies employed kinematic assessment (motion analysis); muscle activity (electromyography, EMG); and/or corticospinal pathway excitability (transcranial magnetic stimulation, TMS) during the reach-to-target task.

### Identification of studies

Studies were assessed as not relevant, probably relevant, or relevant. Title and abstract were screened together. For those studies deemed as either relevant or probably relevant their full texts were then screened (23, 24). Those studies which met the eligibility criteria were included in this review.

### Potential risk of bias

The majority of included studies used observational designs, therefore, the Downs and Black tool was used to assess potential risk of bias (25). The tool was modified by using just the criteria pertinent to potential risk of bias of observational study designs (23, 26). For example: the removal of questions relating to randomization, group allocation, and group concealment (26–28).

### Data extraction

The data extracted were: number of participants, participants' age, time since stroke, reach-to-target task description, use of trunk restraint, upper limb motor ability, kinematic characteristics (e.g., velocity), EMG data (e.g., muscle activity). Some included studies evaluated the effect of an intervention. For these, only the baseline data (pre-intervention) were extracted. For studies in which the published data were unclear or missing then the authors were emailed to request clarification/more details.

### Synthesis

A meta-analysis was undertaken for measures where two or more included studies reported measurement values of the same movement characteristic. A narrative synthesis was performed if there was insufficient similarity across included studies.

If a study included data for multiple reach-to-target tasks one task was selected to be included in the meta-analysis. The task selected was the one most similar to the rest of the studies in the meta-analysis. For example, reaching at a self-paced speed versus fast speeds, tasks in which reaching distances were most similar, and most similar grip (23).

The meta-analysis used the Cochrane Statistical package, RevMan 5.2, to compare the group means and standard deviations of the kinematic characteristics of people after stroke and healthy adult participants. The heterogeneity of data was assessed using the I^2^ statistic and interpreted as low for a value ≤ 25%, high for a value of ≥ 75% and moderate for all values in between (23, 29, 30). If heterogeneity was low a fixed effect model was used; if heterogeneity was moderate or high a random effects model was used (23, 30). The standardized mean difference (SMD) was calculated (23).

## Results

### Identification of studies

**Figure 1:**
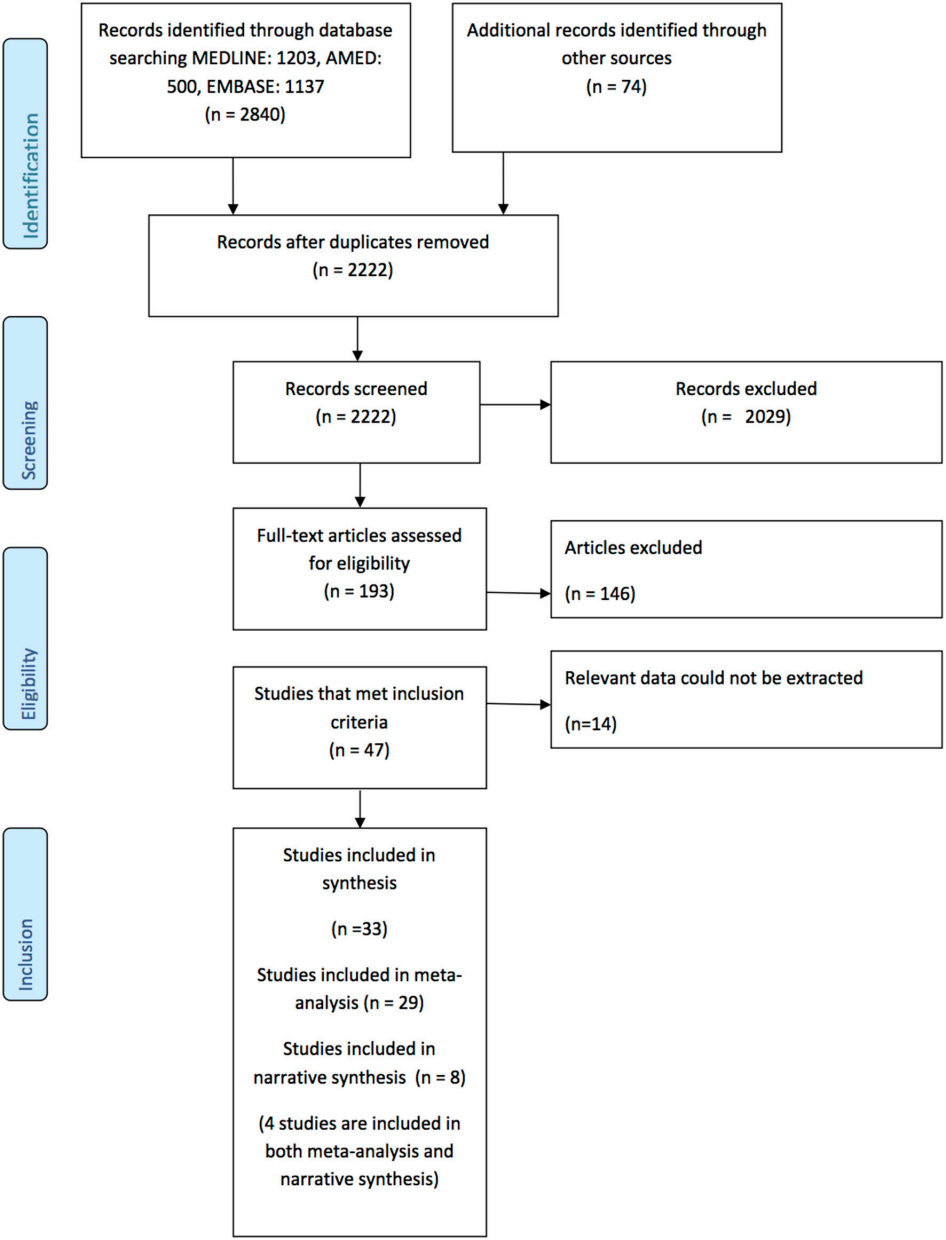
Prisma Diagram detailing the search and processes of identification of studies included in the systematic review.

The flowchart describing the results of the search is provided the PRISMA diagram Figure 1. In summary, 2,222 records were identified after duplicates were removed. Following title, abstract, and full text screening 46 studies met the inclusion criteria, however, 14 were subsequently excluded because the relevant data could not be extracted (9, 31–43). Therefore, there are 33 studies included in the synthesis (5, 7, 13, 14, 16, 44–71). There were two pairs of studies that reported two reaching tasks in the same cohort (16, 63, 67, 68) so participants were only counted once in any particular meta-analysis.

### Included studies

Observational designs were used by 27 of the 32 studies and five studies used experimental designs (5, 45, 47, 48, 61, 69). The included studies investigated reach-to-target with 618 people after stroke and 429 healthy adult participants. The mean number (standard deviation, SD) of individuals per included study was 17.2 ± 9.9 people after stroke and 11.9 ± 9.3 control participants.

### Participants

The mean age (SD) of: people after stroke was 58.4 ± 9.3 years whilst healthy adult participants were a mean (SD) of 54.0 ± 10.0 years. The mean time after stroke, calculated from the data reported, was 25.6 ± 23.1 months. Full details of participants are provided in Tables 2–5 according to the placement of the target in the workspace.

**Table 2:** Characteristics of included studies investigating reach-to-target in multiple anterior areas of the workspace.

**Study**	**Participants**	**Time since stroke**	**Reaching task**	**Kinematic measures**	**Muscle activity measures**
(44)	**S:** 8; Age: 50 ± 16 **C:** 6; Age: 46 ± 16	65 ± 36 months (32–120 months)	Initial target 20 cm from sternum; final targets 35 cm from initial target and 45° to either side of initial target (ipsilateral and contralateral workspace); reach between initial and distal targets Arm support: no Movement speed: fast Trunk restraint: with and without	Movement time Movement smoothness Joint range of motion Compensatory trunk movement Inter-joint coordination	No
(48)	**S:** 9; age 61.4 ± 14.1 **C:** 7; age: 47.2 ± 16.6	3–9 months	Initial target central position then each to a target placed 30 cm from the participant in the ipsilateral, central, and contralateral workspace Arm support: robotic exoskeleton Movement speed: self-selected Trunk restraint: not reported	Execution time Movement smoothness Achievement of target Inward/outward movement time Joint angles Interjoint coordination	Co-contraction ratio of the triceps, biceps, and deltoid (anterior and posterior)
(50)	**S:** 10; age: 71.6 ± 10.4 **C:** 10; age: 68.6 ± 8.7	2.7 ± 1.8 months	Reach to a target in the central, ipsilateral, and contralateral workspace 14 cm from the initial position Arm support: robotic exoskeleton Movement speed: not reported Trunk restraint: not reported	Target accuracy Trajectory or movement straightness	No
(52)	**S:** 16; age: 30–85 **C:** 4; age: 24–39	9 months to 6 years	Reach to targets arranged in 5 rows by 15 columns separated by 12°; reaching to targets was random; initial position thumb on umbilicus Arm support: Wrist splinted Movement speed: self-selected Trunk restraint: yes	Trajectory Velocity Movement smoothness	No
(54)	**S:** 15; age: 59.0 ± 15.4 (30–80) **C:** 12; age: 53.3 ± 17.1	11–101 months	Reach in a physical environment and virtual environment to six targets arranged in a 2 row by 3 column grid in all areas of the anterior workspace in a random order; the distance between participant and the targets were the length of the participant's arm + 50 mm; the target were both above and below shoulder height Arm support: no Movement speed: fast Trunk restraint: not reported	Precision Velocity Trajectory (straightness) Joint range of motion Trunk displacement and rotation Interjoint coordination	No
(55)	**S:** 10; age: 60.9 ± 6.6 **C:** 9; age: 57.8 ± 5.9	2–10 months	Holding a vertical handle (pointer diameter of 3 cm) reach forward 36 cm from initial location and reach lateral 48 cm from initial location. Arm supported: yes, arm brace on smoothed motion plane Movement speed: fast Trunk restraint: not reported (told to move as fast as possible without moving their trunk)	Velocity Reaction time Duration (movement time) Trajectory	EMG of pectoralis clavicular, anterior deltoid, posterior deltoid, biceps, triceps long and lateral head, brachioradialis.
(59)	**S:** 18; age 65.2 ± 9.8 **C:** 8; age 58.6 ± 7.0	2–36 months	Reach to targets (30 mm x 30mm squares) suspended from the ceiling in the ipsilateral, central, and contralateral workspace; target distance was beyond arm's length. The central target was 150 mm above the level of the shoulder; the ipsilateral and contralateral targets were 300 mm to either side of the central target at shoulder height (150 mm lower than the central target). Arm support: no Movement speed: not reported Trunk restraint: no	Velocity Trajectory Joint angles Trunk displacement and rotation Smoothness (number of velocity peaks)	No
(62)	**S:** 7; age: 64.4 ± 13.5 **C:** 7; age: 64.8 ± 11.4	6–37 months	Reach with a wooden dowel to a target at knee height at 160% of arm's length and retrieve a magnetic disc with the dowel in the contralateral and ipsilateral workspace Arm support: no Movement speed: self-selected Trunk restraint: no	Trajectory Joint coordination Joint configuration variance Timing of hand and trunk movement	No
(63)	**S:** 16; age: Left hemisphere damage group: 57 (46–79) right hemisphere damage: 48 (28–78) **C:** 10; age 41 (25–69)	1–27 months	Reach to 9 targets with the palm of the hand; 6 arranged on a table 60% and 90% of arm's length; 3 targets above the table at 90% of arm's length in the central, ipsilateral, and contralateral workspace. Arm support: no Movement speed: self-selected Trunk restraint: no	Velocity Number of velocity peaks (smoothness) Trajectory (curve index)	No
(16)	**S:** 16, age: left hemisphere damage group 57 (46–79), right hemisphere damage group: 48 (28–78) **C:** 10,; age: 41 (25–69)	1–27 months	Reach to 9 targets with the palm of the hand, 6 targets were arranged on the table 60% and 90% of arm's length; 3 targets above the table at 90% of arm's length in the central, ipsilateral, and contralateral workspace. Arm support: no Movement speed: self-selected Trunk restraint: no	Velocity Trajectory (curve index) Trunk displacement Trunk angular rotation, flexion and torsion	No
(13)	**S:** 18; age: 48.8 ± 11.8 **C:** 9 age: 41 (29–71)	0.25 to 15 years	Reach using a pointer to 9 targets; 6 arranged on a table 65% and 90% of arm's length; 3 targets above the table at 90% of arm's length in the central, ipsilateral, and contralateral workspace. Arm support: wrist Splint Movement speed: self-selected Trunk restraint: yes	Velocity Trajectory (curve index) Principle component analysis Precision Joint motion (degrees of freedom of arm joints and scapula)	No
(67, 68) (side of brain damage)	**S:** 14; age 59.5 ± 12.9 (R hemisphere stroke) & 58.9 ± 9.73 9 (L hemisphere stroke) **C:** 6; age 63.8 ± 14.4	R hemisphere stroke 33.2 ± 28.9 months L hemisphere stroke 65.0 ± 33.3	Reach to six targets (3.8 cm sphere) in the ipsilateral and contralateral workspace at a distance of 8 am 16 cm, and 24 cm from the initial start position. Reaches were made without vision. Arm support: Wrist and finger splint to provide support and maintain pointing posture Movement speed: fast Trunk restraint: no Virtual Reality task reaching from the initial positon in front of chest to 6 targets in the ipsilateral and contralateral workspace 8 cm, 16 cm, and 24 cm from initial position. Reaches were made without vision. Arm support: wrist and finger splint to provide support and maintain pointing posture Movement speed: fast Trunk restraint: no	Movement time (a &b) Error/accuracy (a & b) Velocity (a &b) Time to peak velocity (a) Movement distance (a)	No
(69)	**S:** 6; age 71.8 ± 5.4 **C:** 10; age 71.2 ± 5.8	14 to 37 days	Reach to 8 targets placed around a circumference with a radius of 0.14 m. The robot used was the InMotion2 Arm support: Robotics (InMotion 2) Movement speed: not reported Trunk restraint: yes	Trajectory	Muscle synergies

*S, individuals with stroke and c, control participants*.

**Table 3:** Characteristics of included studies investigating reach-to-target task in the contralateral workspace.

**Study**	**Participants**	**Time since stroke**	**Reaching task**	**Kinematic measures**	**Muscle activity measures**
(46)	**S:** 9; age: 54 ± 14 **C:** 9; age: 43 ± 18	2–17 months	Initial target by ipsilateral thigh final target in the contralateral workspace at shoulder height just beyond arms reach; reaches make without vision every 5th trial open eyes to assess final arm position Arm support: no Movement speed: self-selected Trunk restraint: not reported	Movement time Trajectory Error Interjoint coordination# Angular joint motions	No
(14)	**S:** 18; age: 54 ± 17 **C:** 10; age: 43 ± 18	3–17 months	Initial target by ipsilateral thigh final target in the contralateral workspace at shoulder height just beyond arms reach Arm support: no Movement speed: fast Trunk restraint: not reported	Movement time Joint angles Trunk displacement Trajectory Error/accuracy Velocity	No
(14)	S: 20; age: 53.5 ± 16.4 **C:** 10; age: 43.3 ± 18.2	3–17 months	Initial target by ipsilateral thigh final target in the contralateral workspace at shoulder height just beyond arms reach; completed task without vision, every 5th trial open eyes to assess final arm position Arm support: no Movement speed: fast Trunk restraint: not reported	Movement time Coefficient of variability Error Coefficient of variability, Temporal segmentation, Joint ROM Trunk displacement and rotation	No
(45)	**S:** 37; 3 groups age: 1:55.7 ± 15.4 2: 59.1 ± 17.9 3: 64.5 ± 14.1 **C:** 10; age 43.3 ± 18.2	Group 1: 12.1 ± 4.9 Group 2: 11.4 ± 6.3 Group 3: 11.1 ± 5.9 months	Initial target by ipsilateral thigh final target in the contralateral workspace at shoulder height just beyond arms reach Arm support: no Movement speed: fast Trunk restraint: not reported	Movement time Precision (accuracy) Segmentation (smoothness) Velocity variability	No

*S, individuals with stroke and c, control participants*.

**Table 4:** Characteristics of included studies investigating reach-to-target in the ipsilateral workspace.

**Study**	**Participants**	**Time since stroke**	**Reaching task**	**Kinematic measures**	**Muscle activity measures**
(5)	**S:** 8 age 53 ± 8 (SE) **C:** 4; age 28 ± 2.5	46.2 ± 41.5 (12–154) months	Reach from hand on thigh to a target placed 15 mm in front of participant at shoulder height Arm support: no Movement speed: self-selected Trunk restraint: no	Movement time Joint angles Velocity Normalized jerk (measure of smoothness)	No
(56)	**S:** 28; age: 52.2 ± 11.7 **C:** 18; age: 52.1 ± 11.9	19.6 ± 16.3 months	Seated at a table reach to a bell placed in the midsagittal plane shoulder width apart placed at 90 or 125% of arm's length Arm support: no Movement speed: fast Trunk restraint: no	Joint angular changes (shoulder, elbow, trunk)	No
(57)	**S:** 10, age: 58 ± 10 (41–76) **C:** 5 age and sex-matched to stroke participants	>6 months post stroke	In standing reach to a target ball located 5 cm past the outstretched paretic arm of each participant Arm support: no Movement speed: fast Trunk restraint: no	None	EMG activity of: anterior deltoid, middle deltoid, biceps brachii, tibialis anterior, soleus, and sternocleidomastoid Joint ROM
(36)	**S:** 20; age: 60.9 ± 6.1 **C:** 10; age: 61.0 ± 9.0	4.3 ± 2.6 years	Reach to a target with the index finger located at shoulder height within arm's reach Arm support: no Movement speed: fast Trunk restraint: yes	Joint angles Velocity Directness Segmentation Skewness	Muscle activity patterns
(60)	**S:** 8; age 60.5 ± 5 **C:** 10; age 51.5 ± 5	1–10 years	Reaching toward a 0.5 L bottle of water placed in the scapular plane (ipsilateral workspace) at arm's length. Participants had to reach touch the bottle (not grasp) and return. Arm support: no Movement speed: self-selected & fast Trunk restraint: not reported	Velocity Trunk displacement	EMG muscle activity onset
(65)	**S:** 30; age 63.2 ± 12.4 **C:** 30 age matched	29 (6–120) months	Bilateral task of reaching to switches in the ipsilateral/lateral workspace (relative to the reaching arm) 24 cm from the start position and hit a target mounted switch. Arm support: no Movement speed: fast Trunk restraint: not reported	Movement time Reaction time Velocity Trajectory Interlimb coupling	No
(66)	**S:** 11; age 66.1 ± 15.5 **C:** 11; age 51.6 ± 14.5	22.1 ± 13.5 months	Reach to a target 1.3 times arm length in the ipsilateral workspace without vision. There were trials with trunk movement and with trunk movement restrained. Arm support: no Movement speed: not reported Trunk restraint: Trunk free trials and trunk restrained trials	Trajectory Interjoint coordination	No
(70)	**S:** 29; age 63.9 ± 11.7 **C:** 9; age 58.4 ± 13.1	2 time points 8.7 days and 108.7 days after stroke	Reach to touch a 40mm diameter target placed at 90% of arms' length in the ipsilateral workspace at shoulder height Arm support: no Movement speed: fast Trunk restraint: yes	Velocity Trajectory Endpoint error Movement time	Muscle activation patterns EMG muscle activation onset time Modulation ratio
(71)	**S:** 46; age 63.9 ± 13.2 **C:** 10; age 59.1 ± 12.5	9.2 ± 4.2 days	Reach to touch a 40mm diameter target placed at 90% of arms' length in the ipsilateral workspace at shoulder height Arm support: no Movement speed: fast Trunk restraint: yes	Velocity Endpoint error trajectory	No

*s, individuals with stroke and c, control participants*.

**Table 5:** Characteristics of included studies investigating reach-to-target in the central workspace.

**Study**	**Participants**	**Time since stroke**	**Reaching task**	**Kinematic measures**	**Muscle activity measures**
(49)	**S:** 10; age 59.3 ± 9.3 **C:** 10; age 64.1 ± 10.5	7–107 months	Two tasks: reach up or reach down beyond functional arm length 115%. The target height for reaching down was 30 cm from the floor and reaching up was between shoulder and nipple height. Participants had to reach between their lap and the upper/lower targets Arm support: yes Movement speed: fast Trunk restraint: no	Movement time	Muscle coordination via mode vectors and PCA
(51)	**S:** 25; age 64.8 ± 15.9 **C:** 25; age 63.1 ± 16.0	31.5 ± 55 months	Reach to a target 14 cm from the initial position in the central workspace. Arm support: Robotic exoskeleton (REAplan) Movement speed: self-selected Trunk restraint: yes	Accuracy Velocity Trajectory (straightness)	No
(53)	**S:** 11; age: 62.7 ± 11.2 **C:** 8; age: 60.6 ± 6.3	39.4 ± 27.7 (12–94) months	Reach to a target 14 cm from initial position (target displayed on LCD screen) Arm support: deactivated robot exoskeleton Movement speed: self-selected Trunk restraint: yes	Trajectory Peak force and mean force during reaching	EMG amplitude Timing of EMG (muscle) activation
(7)	**S:** 18; age: 67.6 ± 8.1 **C:** 9; age: 57.2 ± 6.7	7–174 months	Reach to a target placed within 80% of arm's length in the participant's midline Arm support: no Movement speed: self-selected & fast Trunk restraint: no	Movement time Velocity Trajectory (index of curvature) Trunk displacement	
(61)	**S:** 14; age: 55.9 ± 11.6 **C:** 14; age: 55.1 ± 9.0	“sub-acute phase”	Unilateral or bilateral task of reaching to a target in front of the body in the central workspace 20 cm from start position Arm support: no Movement speed: fast Trunk restraint: not reported	Movement time Accuracy	
(64)	**S:** 30; **C:** 30 Age: stroke and healthy 67 (4-−86)	29 (6–120) months	Reach forward to hit a switch (in midline) under 3 conditions: unimanual: paretic & non-paretic, and bimanual. Target location was determined such that the action required no more than 15 degrees of elbow extension and 90 degrees of shoulder flexion. Arm support: no Movement speed: fast Trunk restraint: yes	Reaction time Movement time Velocity	

*s, individuals with stroke, c, control participants*.

### Reach-to-target task

The reach-to-target task varied across studies. Heterogeneity was present in: the target distance; target size; target location; reaching speed; trunk restraint; and use of vision for reaching. A description of the reaching tasks, grouped by the location of the target in the workspace, is provided in Tables 2–5. Location of the target in the workspace was considered the pertinent grouping variable because of the expectation of related differences in joint angles, joint trajectories and spatial-temporal patterns of muscle activity.

### Outcome measures

The methods of data collection, kinematic, and EMG outcomes assessed across all studies were diverse. The kinematic characteristics most frequently assessed were: movement time; peak velocity; reach-path-ratio/trajectory; movement smoothness; target accuracy; joint range of motion; and trunk contribution to movement. The EMG-derived assessments most frequently made were: muscle coupling; muscle onset time; and the percentage of muscle used.

### Risk of potential bias

The detailed assessment of risk of potential bias is provided in Table 6. In summary, one study (45) had a low risk of bias across all 13 items of the modified Downs and Black tool (Table 6). There was only one study that was judged to have a high risk of bias for one item (52). This was for participant description. Most of the risk of potential bias was due to unclear reporting of (a) adverse events during the studies and (b) the use of assessors blinded to the intervention/task being investigated.

**Table 6:**
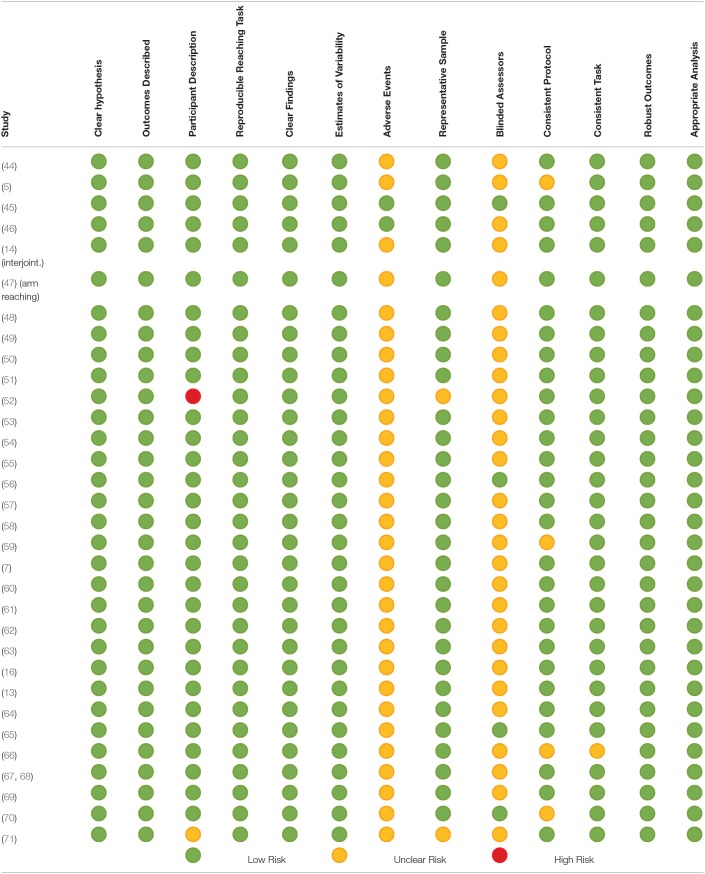
Potential risk of bias of included studies assessed using the modified Down's and Black Tool.

There were seven studies in which the experimental protocol differed for people after stroke and healthy adult participants (9, 61, 63, 69, 72, 73). This was primarily because people after stroke were receiving some rehabilitation and thus had pre/post assessments whereas the healthy adult participants had one assessment only. The reach-to-target task protocols did not differ, thus as the review is utilizing baseline data only this difference in protocol does not impact on the findings and does not contribute to potential bias.

### Synthesis

The synthesis is grouped by workspace location of the target for reach-to-target: central, ipsilateral, contralateral and multiple. Data from 27 of the 32 studies were included in the meta-analysis. The narrative synthesis included data from 8 of the 32 studies.

#### Meta-analysis of kinematic data

**Figure 2:**
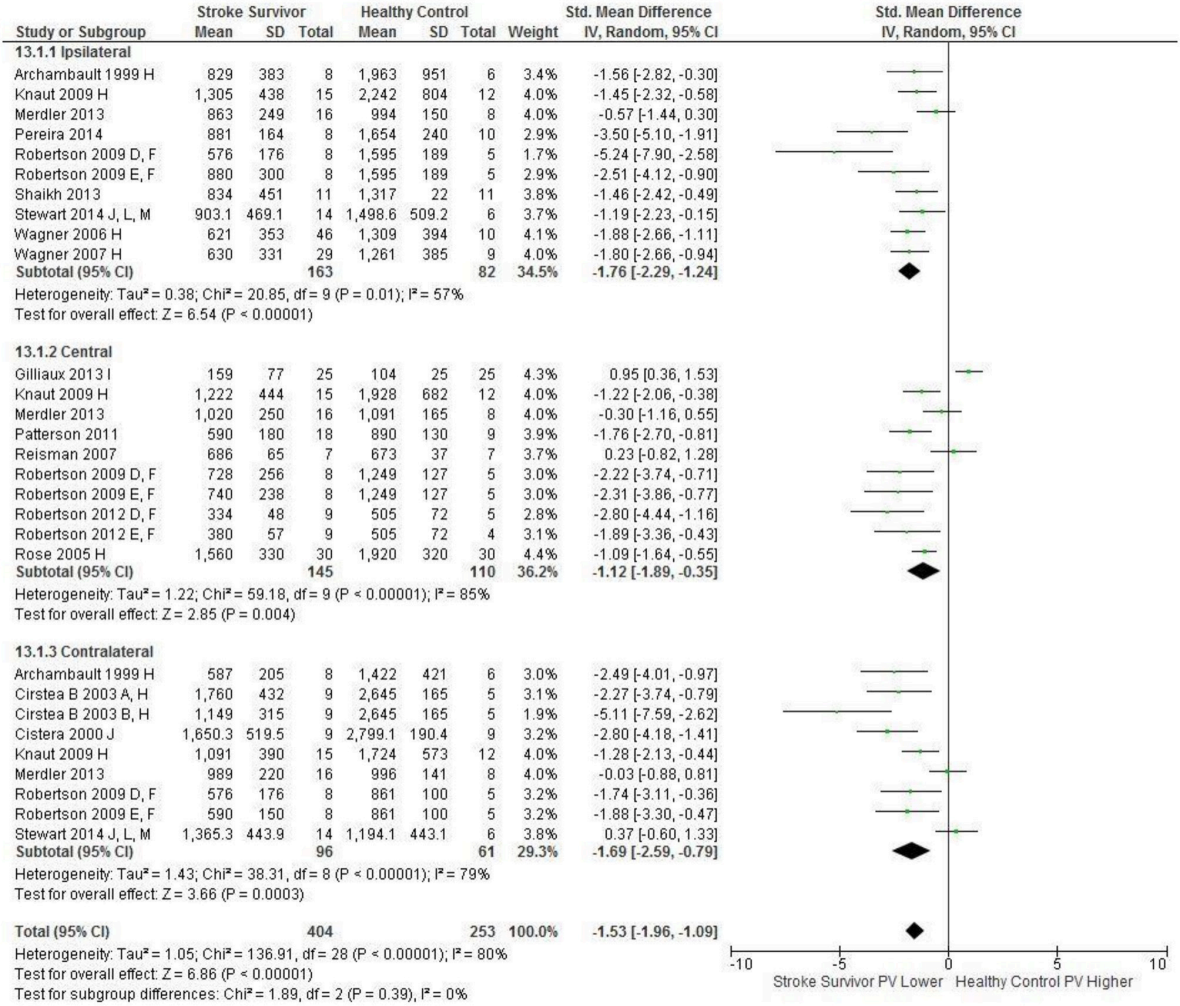
The standardized mean difference (SMD) of peak velocity (mm/s) during reach-to-target in the: ipsilateral, central, and contralateral workspace. D, right hemisphere stroke; E, left hemisphere stroke; F, target placed 90% of arm's length; H, fast speed; I, robotics; J, reaches without vision; L, 24 cm target distance; M, virtual environment.

**Figure 3:**
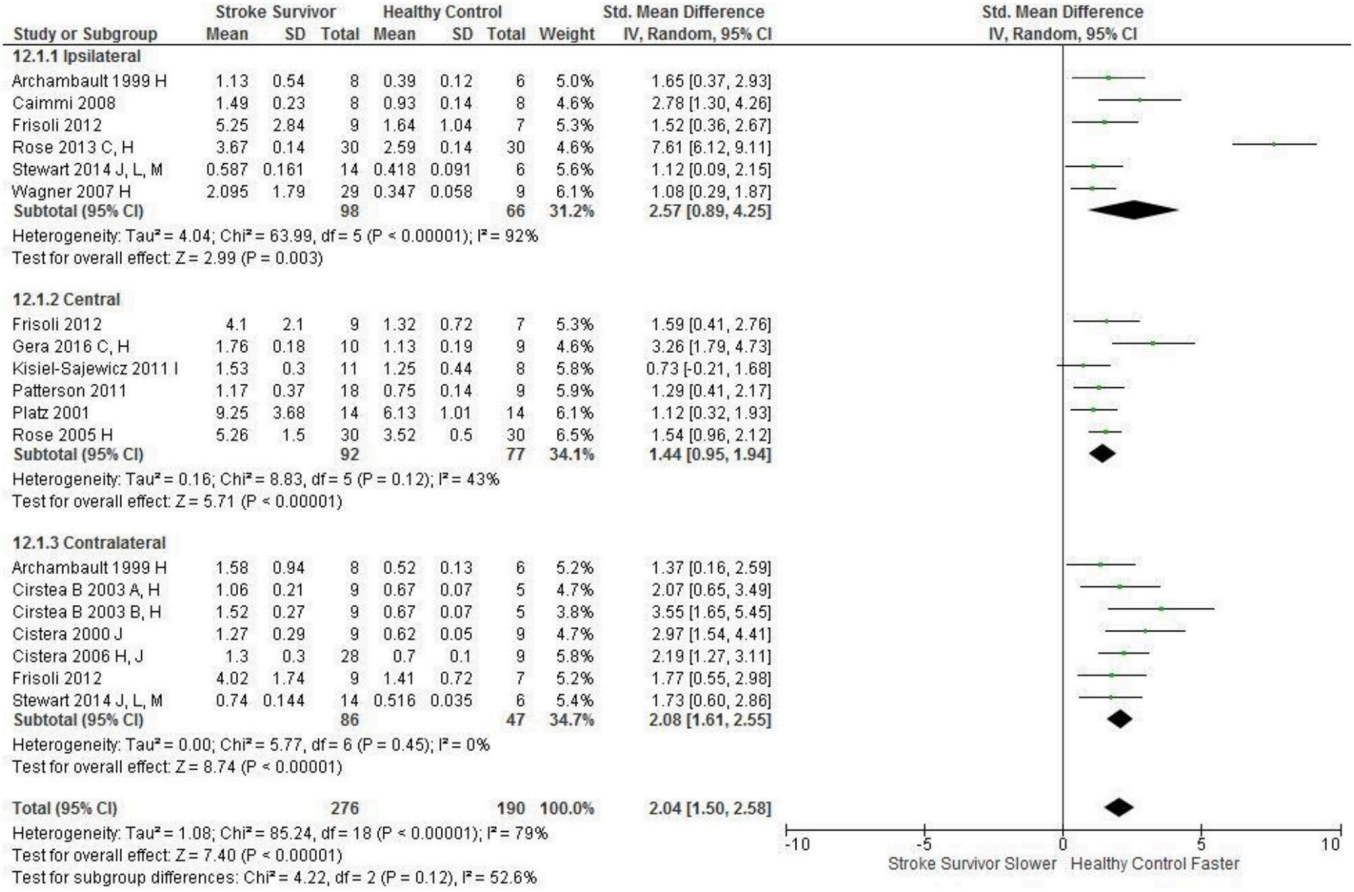
The standardized mean difference (SDM) of movement time (s) during reach-to-target in the: ipsilateral, central, and contralateral workspace. A, mild motor impairment; B, moderate motor impairment; C, bilateral task; F, target placed 90% of arm's length; C, bimanual task; H, fast speed; I, robotics; J, reaches without vision; L, 24 cm target distance; M, virtual environment.

**Figure 4:**
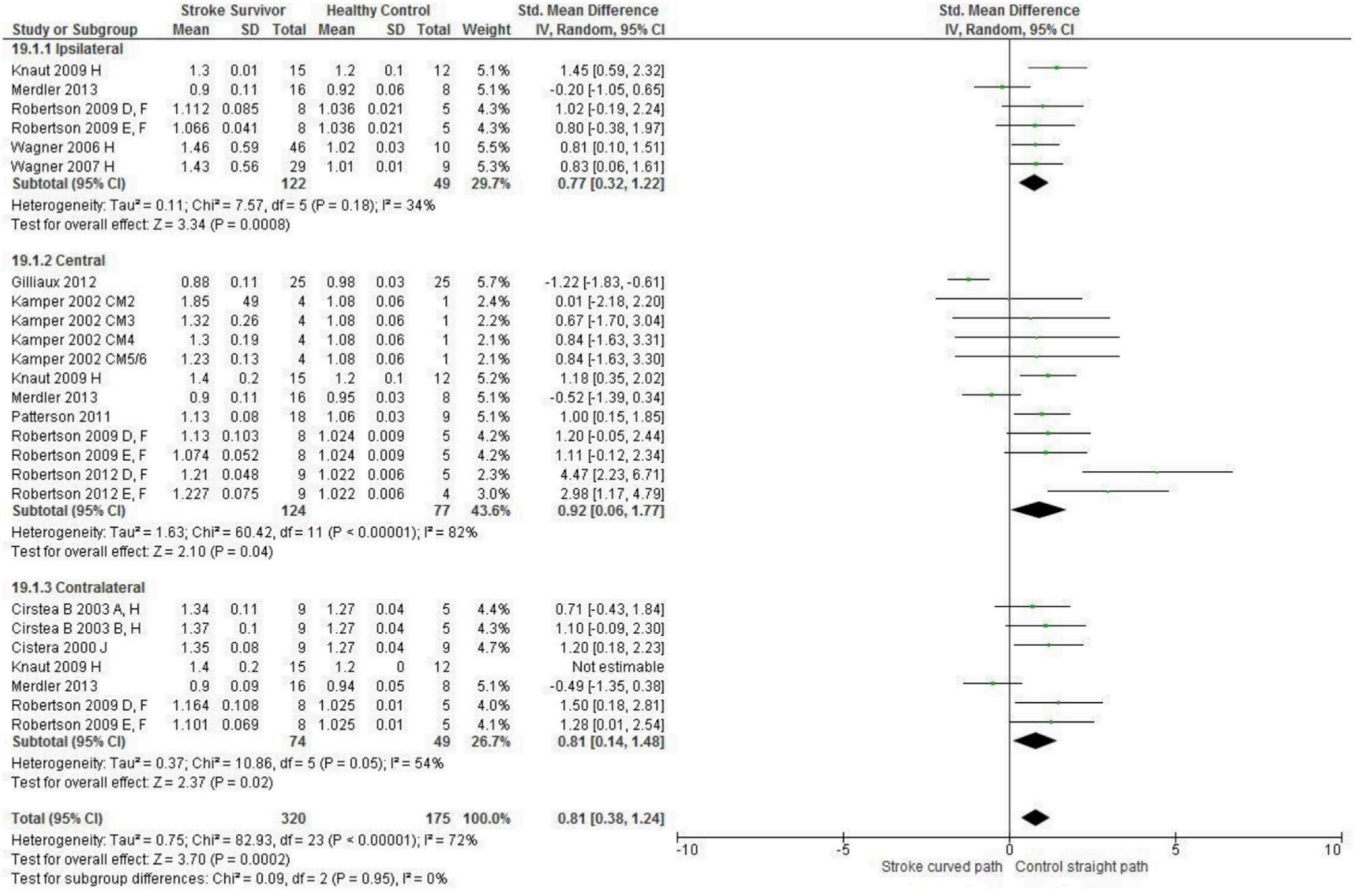
The standardized mean difference (SDM) of reach-path ratio in the: ipsilateral, central, and contralateral workspace. A, mild motor impairment; B, moderate motor impairment; D, right hemisphere stroke; E, left hemisphere stroke; F, target placed 90% of arm's length; H, fast speed; J, reaches without vision; CM, Chedoke-McMaster Stroke Assessment Scale; and corresponding stage (2–6).

**Figure 5:**
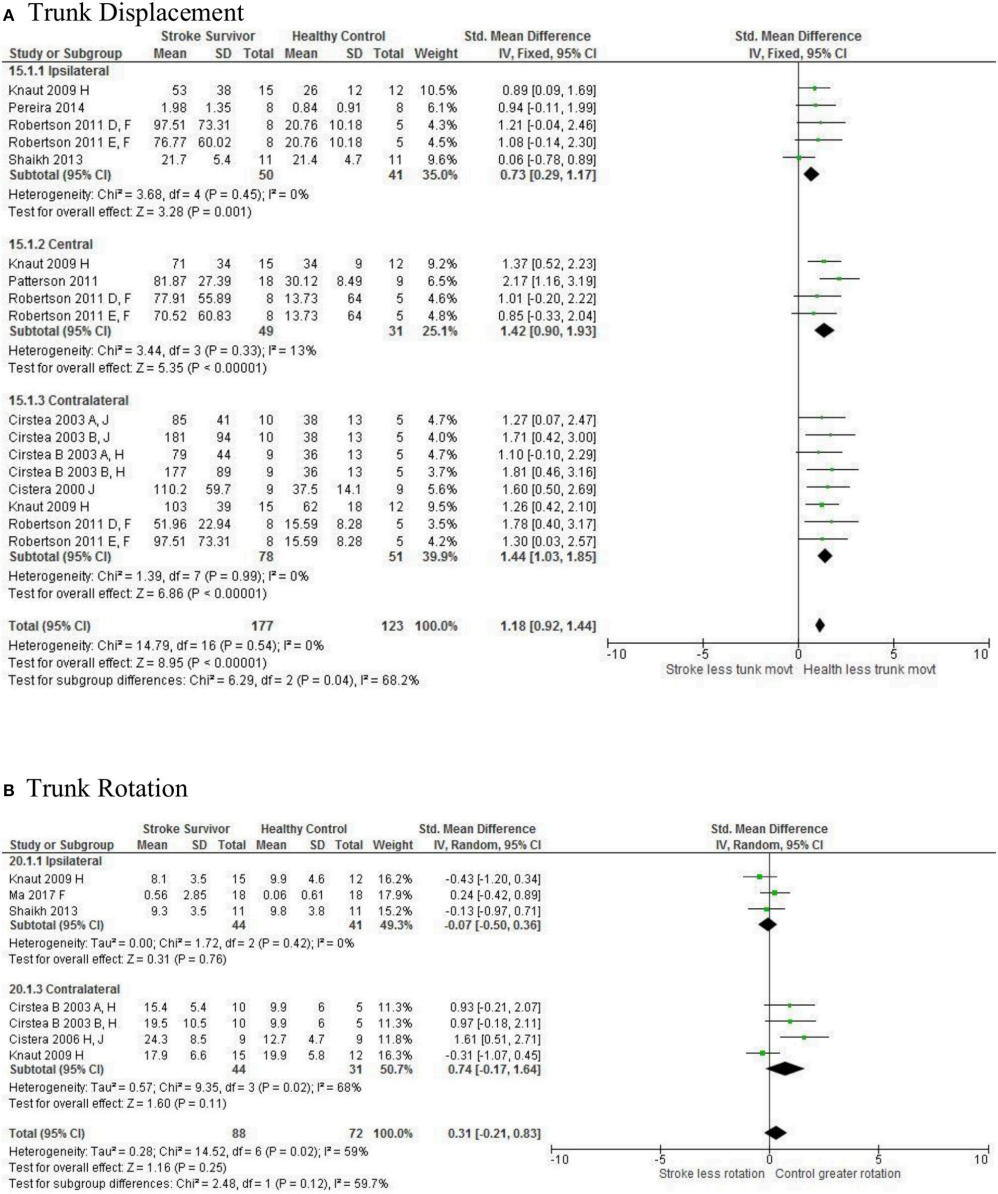
The standardized mean difference (SDM) of trunk displacement (mm) during reach-to-target in the ipsilateral, central, and contralateral workspace. A, mild motor impairment; B, moderate motor impairment; C, bilateral task; D, right hemisphere stroke; E, left hemisphere stroke; F, target placed 90% of arm's length; H, fast speed; LK robotics; J, reaches without vision.

**Figure 6:**
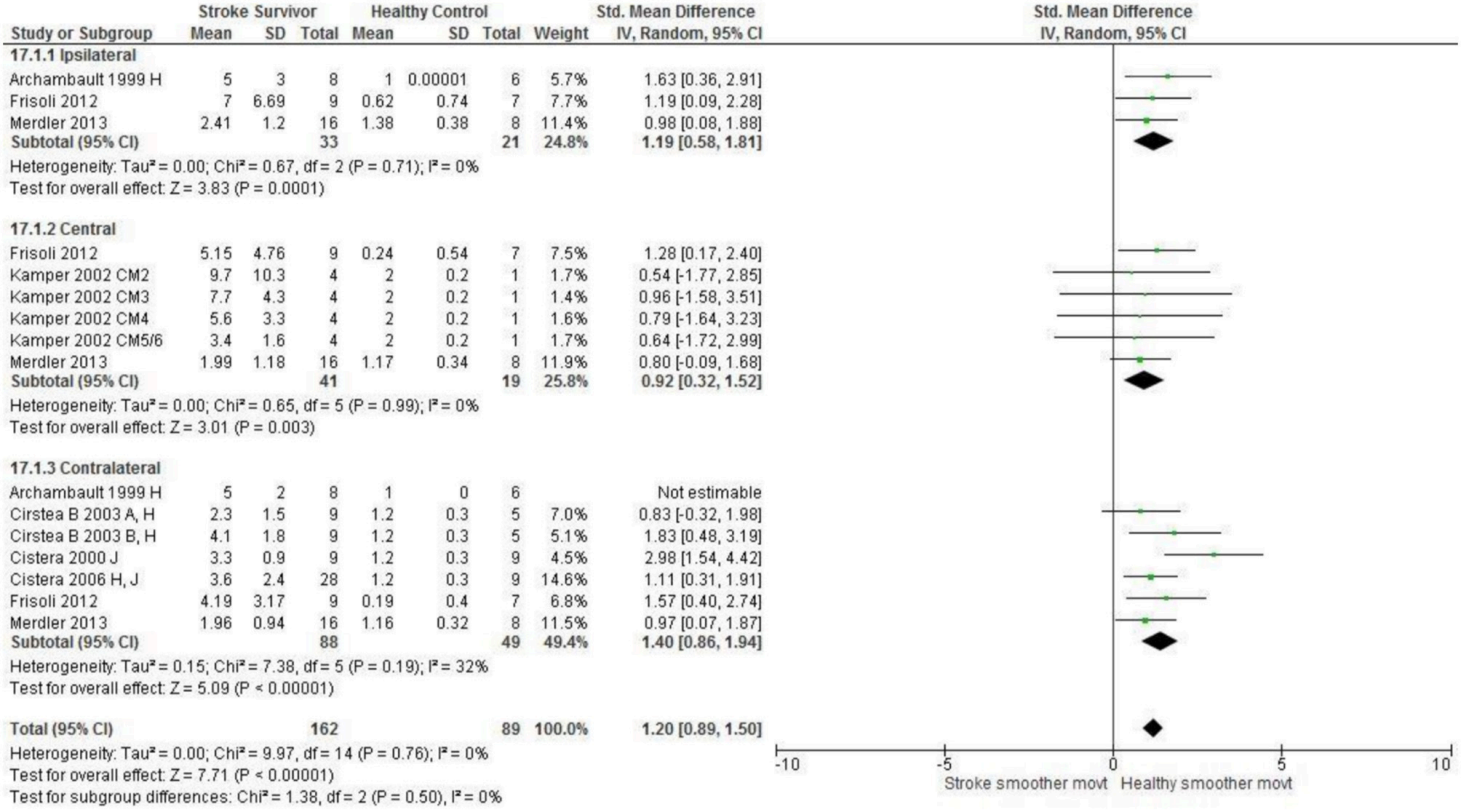
The standardized mean difference (SDM) of movement smoothness during reach-to-target in the ipsilateral, central, and contralateral workspace. A, mild motor impairment; B, moderate motor impairment; H, fast speed; I, robotics; J, reaches without vision; M, virtual environment; CM, Chedoke-McMaster Stroke Assessment Scale; and corresponding stage (2–6).

**Figure 7:**
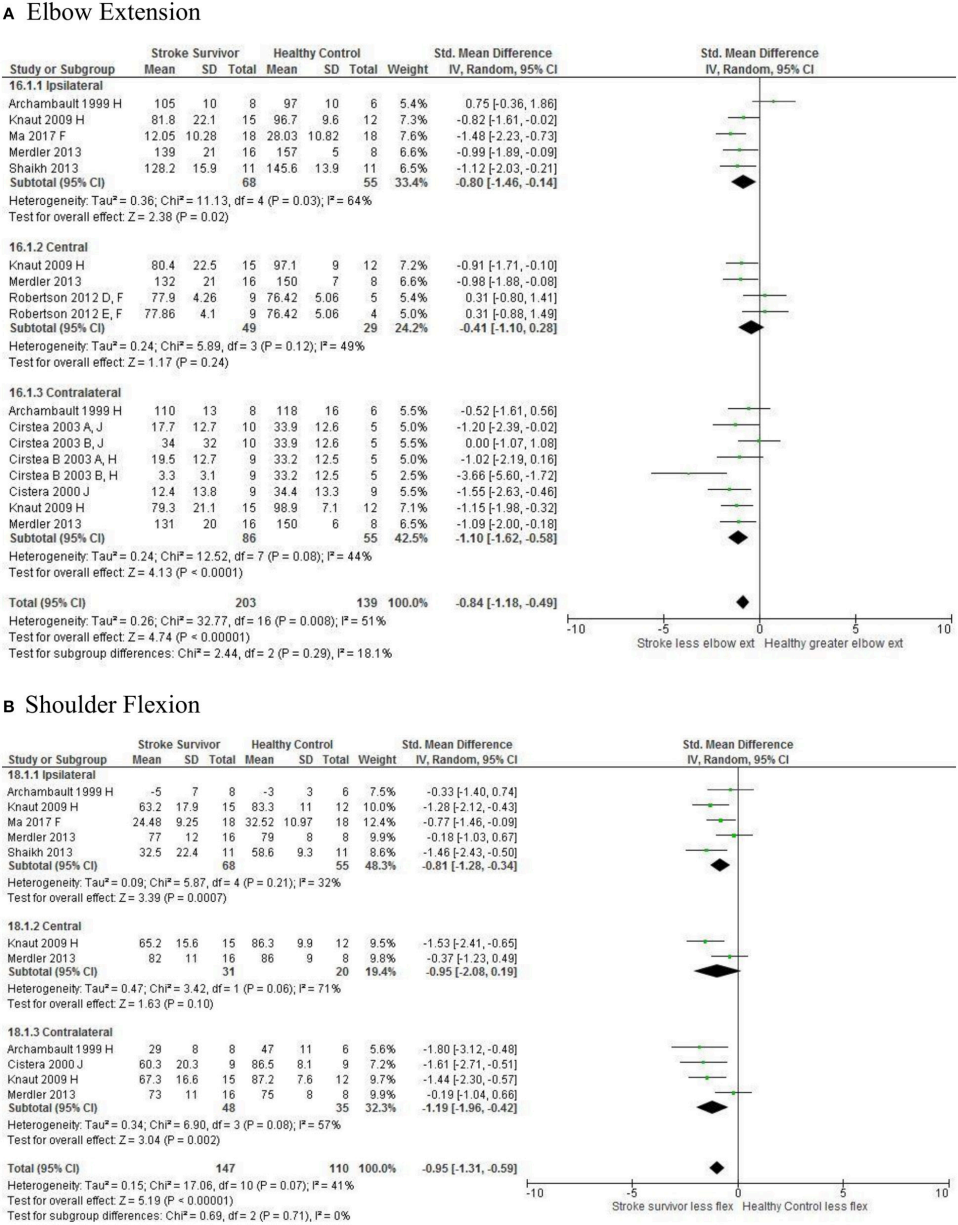
The standardized mean difference (SDM) of joint kinematics in the ipsilateral, central, and contralateral workspace. D, right hemisphere stroke; E, left hemisphere stroke; F, target at 90% of arm's length; H, fast speed; J, reaches without vision.

**Figure 8:**
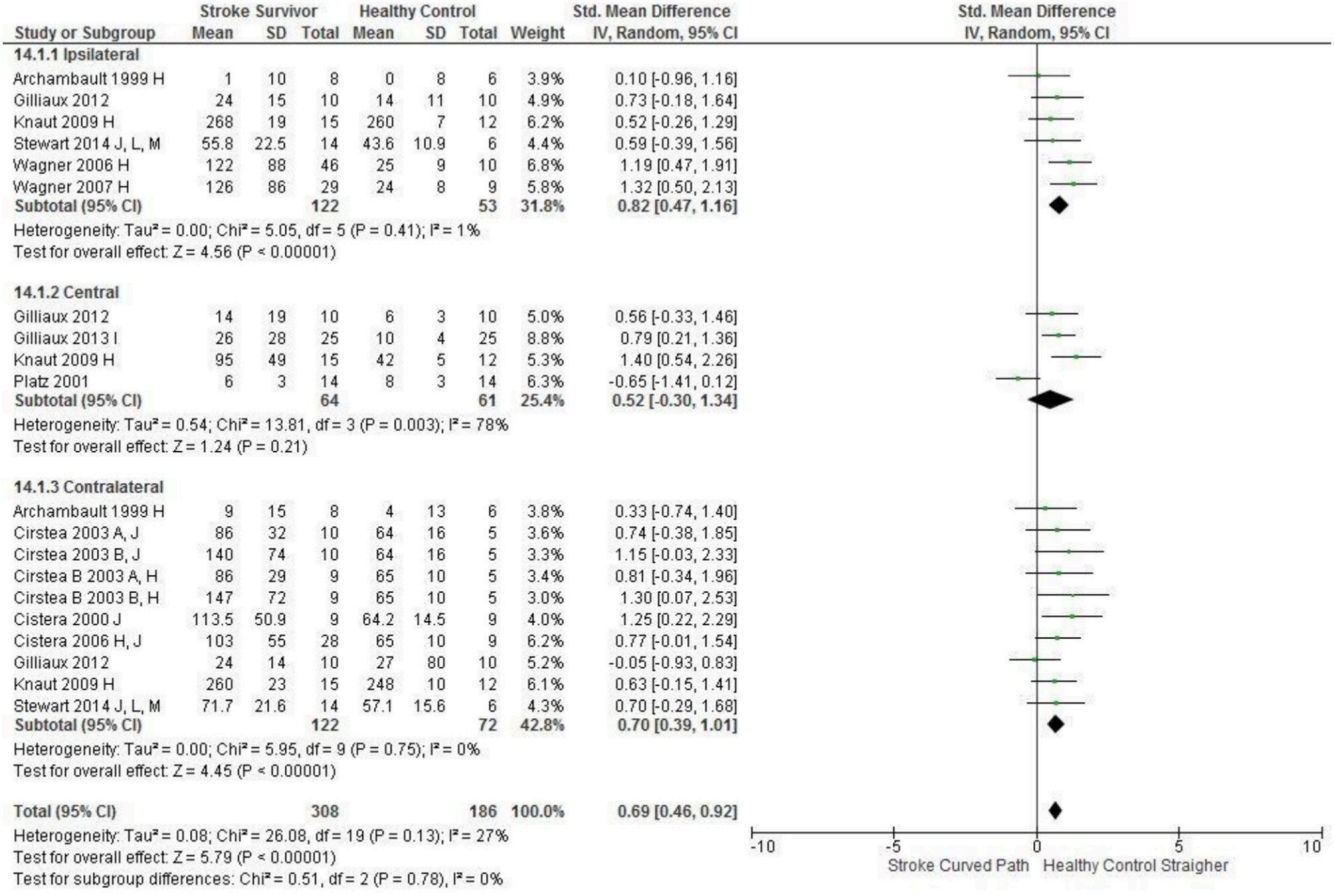
The standardized mean difference (SDM) of accuracy (mm) in the ipsilateral, central, and contralateral workspace. A, mild motor impairment; B, moderate motor impairment; C, bilateral task; H, fast speed; I, robotics; J, reaches without vision; M, virtual environment.

Meta-analysis was possible for the kinematic characteristics of: peak velocity; movement time; reach-path-ratio; smoothness of movement; elbow range of motion (extension); shoulder range of motion (flexion); accuracy; trunk contribution during reaching; and trunk rotation during reaching. Two or more included studies investigated these characteristics. Twenty-six meta-analyses were undertaken. The heterogeneity of the meta-analyses, as measured by the I^2^ statistic, was low (*I*^2^ = ≤ 25%) for 10, moderate (*I*^2^ = 26–74%) for 13, and high (*I*^2^ ≥ 75%) for three (Figures 2–8).

An overview of the meta-analyses is provided in Table 7 and details in Figures 2–8. In summary, 21 of the 26 meta-analyses found significant differences in kinematics between stroke survivors and control participants.

**Table 7:** Summary of the meta-analyses of the kinematic characteristics of reach-to-target.

**Kinematic characteristic and area of workspace**	**Number of participants**	**SMD [95% CI]**	**Stroke participants compared to control participants**
Peak velocity: Central	Stroke = 145, Control = 110	−1.12 [−1.90, −0.35]*	↓
Peak velocity: Ipsilateral	Stroke = 163, Control = 82	−1.76 [−2.29, −1.24]*	↓
Peak velocity: contralateral	Stroke = 96, Control = 61	−1.69 [−2.59, −0.79]*	↓
Movement time: Central	Stroke = 92, Control = 77	1.44 [0.95, 1.94]*	↑
Movement time: Ipsilateral	Stroke = 98, Control = 66	2.57 [0.89, 4.25]*	↑
Movement time: Contralateral	Stroke = 86, Control = 47	2.08 [1.61, 2.55]*	↑
Reach path tatio: Central	Stroke = 124, Control = 77	0.92 [0.06, 1.77]*	↑
Reach path ratio: Ipsilateral	Stroke = 122, Control = 49	0.77 [0.32, 1.22]*	↑
Reach path ratio: Contralateral	Stroke = 74, Control = 49	0.81 [0.14, 1.48]*	↑
Trunk contribution: Central	Stroke = 49, Control = 31	1.42 [0.90, 1.93]*	↑
Trunk contribution: Ipsilateral	Stroke = 50, Control = 41	0.73 [0.29, 1.17] *	↑
Trunk contribution: Contralateral	Stroke = 78, Control = 51	1.44 [1.03, 1.85]*	↑
Smoothness of movement: central	Stroke = 41, Control = 19	0.92 [0.32, 1.52]*	↓
Smoothness of movement: Ipsilateral	Stroke = 33, Control = 21	1.19 [0.58, 1.81]*	↓
Smoothness of movement: contralateral	Stroke = 88, Control = 49	1.40 [0.86, 1.94]*	↓
Elbow extension: Central	Stroke = 49, Control = 29	−0.41 [−1.10, 0.28]	↔
Elbow extension: Ipsilateral	Stroke = 68, Control = 55	−0.80 [−1.46, −0.14]*	↓
Elbow extension: Contralateral	Stroke = 86, Control = 55	−1.10 [−1.62, −0.58]*	↓
Shoulder flexion:Central	Stroke = 31, Control = 20	−0.95 [−2.08, 0.19]	↔
Shoulder flexion: Ipsilateral	Stroke = 68, Control = 55	−0.81 [−1.28, −0.34]*	↓
Shoulder flexion: Contralateral	Stroke = 48, Control = 35	−1.19 [−1.96, −0.42]*	↓
Accuracy: Contralateral	Stroke = 122, Control = 72	0.70 [0.39, 1.01]*	↑
Accuracy: Ipsilateral	Stroke = 122, Control = 53	0.82 [0.47, 1.16]*	↑
Accuracy: Central	Stroke = 64, Control = 61	0.52 [−0.30, 1.34]	↔
Trunk rotation: Contralateral	Stroke = 44, Control = 31	0.74 [−0.17, 1.64]	↔
Trunk rotation: Ipsilateral	Stroke = 44, Control = 41	−0.07[−0.50, 0.36]	↔

A fixed effect model was used if I^2^ ≤ 25%, and a random effects model was used if I^2^ > 26 %. SMD, standardized mean difference; 95%CI, 95% confidence intervals; * indicates significant difference in SMD between individuals with stroke and control participants; ↑, significantly greater in individuals with stroke; ↓, significantly decreased in individuals' with stroke; and ↔, no differences between individuals' with stroke and control participants.

The SMD (95% CIs) for the significant differences in kinematic characteristics between people after stroke and healthy adult participants ranged from: −1.76 (−2.29, −1.24) for peak velocity in the ipsilateral workspace to 2.57 (0.89, 4.25) for movement time in the ipsilateral workspace. *Individuals with stroke demonstrated lower peak velocities and longer movement times in all areas of the workspace (Figures* *2*, *3**). A more curved reach-path-ratio associated with less efficient reaching was demonstrated by individuals with stroke (Figure* *4**) as well as less smooth more segmented movement due to a greater number of velocity peaks in all areas of the workspace (Figure* *6**). Individuals with stroke demonstrated greater trunk displacement during reaching (Figure* *5**), less upper limb range of motion in all areas of the workspace (Figure* *7**) and reduced reaching accuracy (Figure* *8**)*.

The non-significant differences between people after stroke and healthy adults for kinematics during reaching were: elbow extension in the central workspace SMD = −0.41 [−1.10, 0.28]; target accuracy in the central workspace SMD = 0.52 [−0.30, 1.34]; trunk rotation in the contralateral workspace SMD = 0.74 [−0.17, 1.54], trunk rotation in the ipsilateral workspace SMD = −0.07 [−0.50, 0.36]; and shoulder flexion in the central workspace SMD = −0.95 [−2.08, 0.19].

#### Narrative synthesis of muscle activity data

The muscles most frequently investigated were the: triceps, biceps, deltoid (anterior, posterior, and middle), trapezius, pectoralis, and latissimus dorsi. Six studies investigated interaction between muscle pairs (48, 53, 69, 70, 72, 74). Also investigated were muscle activation patterns (58, 60), muscle timing (57, 60), and the percentage of muscle activity used in relation to the maximal voluntary contraction (MVC) (55, 58, 69, 70).

There were comparable findings across studies. For example, compared to healthy adult participants the people after stroke used a greater percentage of MVC (58, 70), higher background muscle activity (55, 69), a reduced level of coherence between antagonistic muscle pairs (48, 53, 74), and prolonged co-contraction between muscles after achieving the task (55).

There were also differences between studies. For example, delayed onset of muscle activation in stroke survivors compared to healthy adult participants (57, 60, 74), contrasts with findings of no significant difference between the two groups (53).

The synthesis also suggests that just examining one aspect of muscle activity might not be sufficient for identification of potential therapy targets after stroke. For example, people after stroke and healthy adult participants were found to utilize a similar number of muscle synergies during reaching (69, 72). But, a notable difference was that healthy adult participants during arm abduction and flexion recruited the anterior deltoid and pectoralis major whereas people after stroke recruited additional muscle of the brachioradialis and brachial (69).

## Discussion

The meta-analysis reported here found that people after stroke, compared with healthy adult participants, demonstrate: longer movement time, decreased peak velocity, greater trunk contribution, less smooth movement, and a more curved reach path when performing reach-to-target in all areas of the workspace. Furthermore, people after stroke exhibit less accurate reaches and decreased elbow extension reaching *to objects* in the ipsilateral and contralateral workspace; and less shoulder flexion when reaching in the contralateral and ipsilateral workspace. *Object location in the workspace influenced joint range of motion and target accuracy such that there were no differences between individuals with and without stroke in the central workspace*. These kinematic elements of movement skill are potential targets for rehabilitation therapy.

The narrative analysis reported here suggests that compared with healthy adult participants, people after stroke performing reach-to-target: use a greater percentage of MVC, have higher background muscle activity, and decreased coherence between muscle pairs. Meta-analysis was precluded by heterogeneity between included studies therefore caution needs to be used in considering these elements of movement skill as potential targets for rehabilitation therapy.

*The meta-analysis finding reported here are applicable to individuals with stroke that exhibit similar levels of motor function to those individuals within the studies e.g., have the motor control to reach and point (mild to moderate upper limb deficits)*.

### Comparison with earlier published findings

Interpretation of the present findings needs to be made considering the risk of potential bias of included studies. Most items were assessed as low risk; however, there was one area of one study assessed as high risk. Overall there was unclear reporting of both adverse events and blinded assessment for most included studies. The influence of these indications of risk of potential bias is debatable. It is reasonable to propose that reporting adverse events is irrelevant to this review because most included studies did not investigate an intervention and for those that did, only the baseline measures were included. It is also possible that unclear reporting of blinded assessment is not directly relevant to the results of this systematic review as measures derived from kinematic assessment and EMG are objective. However, the risk of potential bias from unclear reporting of blinded assessment remains if the same researcher conducted the assessments and those conducting processing and statistical analysis of the movement data. So, caution remains in respect of unclear reporting of blinded assessment. Otherwise, there is mostly low risk of potential bias and therefore the meta-analysis results are considered to be strong.

The identified kinematic differences during reach-to-target are mostly in accordance with previous narrative reviews (4, 19, 20). However, the study reported here is the first-ever meta-analysis of reach-to-target, using a systematic literature search unlike two of the earlier reviews (4, 20) and employed a systematic approach for reviewers to identify relevant studies and extract data unlike any of the earlier reviews (4, 19, 20). The results therefore are less likely to be confounded by reviewer bias than the earlier reviews. The results reported here provide the kinematic differences, and their variances, during reach-to-target performed by people after stroke and healthy adult participants. Objective reference values that could be used for target setting for upper limb rehabilitation after stroke can also be derived from this review. Consequently, the review reported here has provided additional knowledge to that provided in the earlier narrative reviews. Especially as the earlier reviews examined a variety of tasks involving reaching (19); reach-to-grasp rather than reach-to-target; and did not specify the aspects of reaching that were reviewed. This difference between reviews is important as it has been known for some time that kinematic characteristics differ between different reaching tasks (21, 22, 75).

Unlike the earlier narrative reviews (4, 19, 20) the review has examined EMG-derived measures of reach-to-target. It is possible that reduced coherence between muscles contributes to the kinematic differences between individuals with and without stroke such as reduced peak velocity and decreased movement smoothness. Suchc an association has been found between a reduced number of muscle synergies and reduced gait speed after stroke which was subsequently correlated with walking dysfunction (76).

This review found conflicting findings for timing of muscle activation. One study identified no difference in muscle onset time in comparison to control participants (53). Whereas, another found that individuals with stroke have delayed muscle onset/activation (57, 60, 74). Clearly this is an area for future research.

Interestingly 12 reach-to-target studies included reaching into the contralateral workspace. Yet healthy adults, when given the option to use their preferred arm to reach to target in any area of the workspace, utilize ipsilateral reaches rather than contralateral reaches during spontaneous activity (left arm for left targets, and right arm for (77) right targets) (34, 78, 79). Potential explanations for preferred ipsilateral reaches are that contralateral reaches are less biomechanically efficient thus require greater energy (79). *Workspace location had minimal influence on the differences in kinematics between individuals with stroke and control with there being consistent significant differences in all areas of the workspace. However, in the central workspace there were no differences in shoulder/elbow range of motion or accuracy between individuals with and without stroke. This could be due to the joint combinations needed to reach to the central workspace (e.g. elbow extension with shoulder adduction) are part of the flexor synergy in individuals with stroke, an often used movement pattern (77)*.

### Strengths and limitations

The studies included in the systematic review were heterogeneous, for example: the reaching task; movement speed; object location; use of trunk restraint; upper limb motor ability of individuals with stroke; and varied time since stroke. The I^2^ statistic demonstrated that of the 26 meta-analyses three meta-analyses had high heterogeneity (*I*^2^ ≥ 75%) reach-path-ratio (central workspace), peak velocity (central workspace), and movement time in the ipsilateral workspace. The remaining twenty three meta analyses exhibited low (10/26) and moderate heterogeneity (13/26) (23, 30). Evaluation of the forest plots demonstrates that many of the confidence intervals are overlapping and the mean differences fall on the same side of the line of no effect (23, 30) suggesting the studies are comparable. However the possibility remains that combing heterogeneous studies with in a meta-analysis could be a limitation as the findings may be biased (23).

There are two additional potential limitations to this review. First, limitation of the search to articles published in the English language. However, a strength is that the search strategy was robust and carried out in multiple data-bases. The second limitation is that participants with stroke had to have sufficient upper limb motor function to complete the reaching task, so, the findings may not be applicable to those with severe paresis.

## Conclusion

This first-ever meta-analysis of the kinematics of reach-to-target by people with stroke and healthy adults performing reach-to-target found 21 elements that could provide targets for impairment-orientated therapy for better upper limb recovery. *Of the kinematic characteristics, object location influenced joint range of motion and target accuracy*.The findings also quantify the differences which should inform measurement of the efficacy of rehabilitation. Subsequent studies need to investigate whether tailoring therapy at the identified differences reported here, does enhance upper limb recovery after stroke.

## Author contributions

KC led the conception, design, analysis and interpretation of this systematic review, prepared the initial drafts of the report, provided approval for publication of the content and agreed to be accountable for all aspects of the work. NK made substantial contributions to the conception, design, analysis and interpretation of this systematic review, contributed to drafts of the report, provided approval for publication of the content and agreed to be accountable for all aspects of the work. AC made substantial contributions to the conception, design, analysis and interpretation of this systematic review, contributed to drafts of the report, provided approval for publication of the content and agreed to be accountable for all aspects of the work. VP made substantial contributions to the conception, design, analysis and interpretation of this systematic review, contributed to drafts of the report, prepared the final version of this report, provided approval for publication of the content and agreed to be accountable for all aspects of the work.

### Conflict of interest statement

The authors declare that the research was conducted in the absence of any commercial or financial relationships that could be construed as a potential conflict of interest.
